# Analysis and Design of Fiber Microprobe Displacement Sensors Including Collimated Type and Convergent Type for Ultra-Precision Displacement Measurement

**DOI:** 10.3390/mi15020224

**Published:** 2024-01-31

**Authors:** Yisi Dong, Jinran Zhang, Chen Zhang, Haijin Fu, Wenwen Li, Wenrui Luo, Pengcheng Hu

**Affiliations:** 1 Center of Ultra-Precision Optoelectronic Instrument, Harbin Institute of Technology, Harbin 150080, China; dongyisi2013@163.com (Y.D.); 23s001044@stu.hit.edu.cn (J.Z.); haijinfu@hit.edu.cn (H.F.); spark20000804@163.com (W.L.); luowenrui2023@163.com (W.L.); hupc@hit.edu.cn (P.H.); 2 Key Lab of Ultra-Precision Intelligent Instrumentation, Ministry of Industry and Information Technology, Harbin Institute of Technology, Harbin 150080, China

**Keywords:** fiber laser interferometer, microprobe sensors, Michelson laser interference

## Abstract

In this paper, a fiber optic microprobe displacement sensor is proposed considering characteristics of micro-Michelson interference structure and its components. The principal error of micro Fabry–Perot interferometric structure is avoided, and high-precision interferometric displacement measurement is realized. The collimated microprobe and convergent microprobe are analyzed, simulated, and designed for the purposes of measuring long-distance displacement and small spot rough surface, respectively. The core parameters of the probes’ internal components are mapped to coupling efficiency and contrast of the sensor measurements, which provides a basis for the probes’ design. Finally, simulation and experimental testing of the two probes show that the collimated probe’s working distance and converging probe’s tolerance angle can reach 40 cm and ±0.5°, respectively. The designed probes are installed in the fiber laser interferometer, and a displacement resolution of 0.4 nm is achieved.

## 1. Introduction

As an important and indispensable technology and core strategy in the field of ultra-precision displacement measurement, laser interferometer is also developing in the direction of high-integration, small-volume, and ultra-precision embedded on-line measurement [1,2,3]. Compared with the traditional laser interferometer, the fiber laser interferometer has significant advantages, such as easy installation and adjustment, isolation of thermal pollution, and easy embedded measurement realization. There are four main categories of fiber optic interferometers: fiber optic Michelson interferometers [4,5,6,7], fiber optic Mach–Zehnder (M-Z) interferometers [8], fiber optic Sagnac interferometers [9], and fiber optic Fabry–Perot (F-P) interferometers [10,11], among which only fiber optic Michelson interferometers and fiber optic Fabry–Perot interferometers are capable of performing embedded displacement measurements. For the fiber optic Michelson laser interferometer, the stability and anti-interference are not optimized because the reference and measurement light inside the fiber are easily affected by the external environment. Even though researchers have studied the use of dual interferometers to compensate for the common optical path [12], the method is still not practical. The fiber optic Fabry–Perot laser interferometer is more resistant to interference because the reference and measurement lights are in a common optical path and the optical fiber is only used for transmission. Therefore, the sensing method using the F-P cavity formed by the fiber end-face and the target object has received widespread attention.

The initial version of fiber optic F-P interferometer was composed of a single-mode fiber end-face and a target because the beam out of the fiber presented a cone angle dispersion; therefore, light return efficiency was very low and, as a result, it was difficult to carry out long-distance measurements and its probing distance was generally around 1 mm [13]. Some researchers processed the fiber structure to achieve a tapered core structure in order to reduce the outflow light beam dispersion angle, increase the fiber optic microprobe detection distance, and ultimately achieve an 80 mm working distance [14]. In order to further increase the micro-sensing probes’ measurement distance, a method was proposed to match the single-mode optical fiber parameters using convex and variable refractive index lenses, which ultimately resulted in better beam collimation and increased the detection distance to 30–40 cm [15,16,17,18]. However, the above structure approximates the multi-beam interference into a two-beam interference, an approximation that is acceptable in some sensing fields where high precision is not required. In the field of ultra-precision displacement sensing, however, the resulting higher-order cyclic principle error seriously affects the accuracy of laser interference displacement measurement.

To address the lack of high-precision micro-sensing probes, proper design solutions, and models, we propose a fiber optic Michelson-type microprobe interference structure in this study. The creation of a multi-beam interference principle error is avoided through application of the two-beam interference principle for micro-sensing probes. First, a theoretical model was developed for the fiber optic Michelson microprobe. A mapping relationship between the probe’s structural parameters and working distance and tolerance angle was then developed. The design parameters of the collimated micro-sensing probe were finally studied. Moreover, we expanded the convergent microprobe, which is designed to meet the measurement needs of small spots and rough reflective surfaces, thus facilitating the application of microprobe laser interferometers. In Section 2, we introduce the fiber optic microprobe laser interferometer, discuss the error analysis of the traditional F-P microprobe, and then introduce the structure and design considerations of Michelson-type microprobes. In Section 3, we discuss the experimental and simulation results associated with the collimated and convergent microprobes. Lastly, the displacement measurement results for these microprobes are presented.

## 2. Design of Two Michelson-Type Microprobe Sensors

### 2.1. Fundamentals of Fiber Optic Microprobe Laser Interferometry

**Figure 1:**
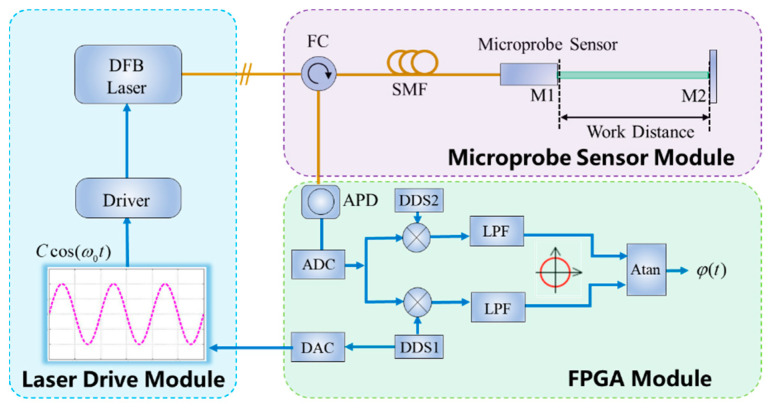
Schematic diagram of fiber optic microprobe laser interferometer measurement (note: DFB: Distributed Feedback Laser; FC: Fiber Circulator; SMF: Single-Mode Fiber; APD: Avalanche Photodetector; DSS: Direct Digital Synthesizer; ADC: Analog-to-Digital Converter; DAC: Digital-to-Analog Converter; LPF: Low-Pass Filter; Atan: the inverse tangent algorithm; M1/2: Mirrors 1 and 2).

The measurement principle of the fiber optic microprobe laser interferometer is as follows: the FPGA signal processing board generates a sinusoidal modulation signal through the Direct Digital Synthesis (DDS) technique. The drive current is then applied to the distributed feedback semiconductor laser (DFB) so that the light’s output wavelength is modulated by a high frequency (Figure 1). The output light propagates through the optical fiber as well as the circulator and interferes with the micro-sensing probe with the following expression:

(1) $$I=I_{r}+I_{s}+2\sqrt{I_{r}I_{s}}\cos\left(\varphi_{0}+\varphi_{x}\right),$$

where *I*_r_ denotes the intrinsic reference light intensity, *I*_s_ represents the measured light intensity, $\varphi_{0}$ is the initial phase, and $\varphi_{x}$ is the phase difference corresponding to the distance to be measured.

The interfering signals are again propagated through the circulator and optical fiber and are received by photodetectors, such as avalanche photodiodes (APD), and converted into the corresponding current signals:

(2) $$S\left(t\right)=K(I_{r}+I_{s})+2K\sqrt{I_{r}I_{s}}\cos\phi =A+B\cos\left(\varphi_{0}+\varphi_{x}\right),$$

where *K* denotes the photoelectric conversion coefficient of the photodetector, $A=K(I_{r}+I_{s})$ is the DC bias, and $B=2K\sqrt{I_{r}I_{s}}$ is the amplitude of the AC signal carrying the displacement information.

Based on the interferometric displacement measurement, the relationship between the measurable phase and displacement is given by:

(3) $$\varphi_{x}=\frac{4\pi nL_{x}}{\lambda },$$

where *n* denotes the air refractive index, *L*_x_ is the displacement to be measured, and *λ* denotes the laser wavelength.

After the photoelectric conversion, the signal *S*(*t*) is first converted from analog to digital in the signal processing board, and then the phase is demodulated in FPGA using the Phase Generated Carrier (PGC) algorithm [19,20] so that the final displacement can be further calculated.

### 2.2. Analysis of High-Order Nanoscale Errors in F-P-Type Microprobes

**Figure 2:**
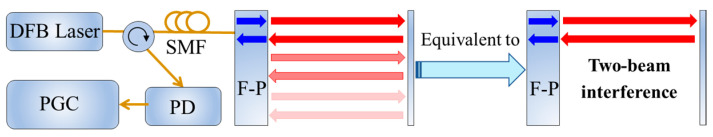
Schematic diagram of fiber optic F-P microprobe displacement sensing (note: PD: Photodetector; PGC: Phase Generation Carrier).

To obtain the interference signal as discussed in Section 2.1., the fiber optic Fabry–Perot micro-sensing probe has been widely developed. This probe has a common optical path between the reference and measuring lights. This microprobe has high measurement accuracy, a small size, and a simple structure, and it is commonly applied in the field of precision displacement measurement. The structure and measurement principle of the fiber optic Fabry–Perot micro-sensing probe laser interferometer is schematically shown in Figure 2. An expression for the interference signal associated with the fiber optic F-P microprobe is given by:

(4) $$I_{\mathrm{FP}}=I_{0}\cdot \frac{r_{1}^{2}+r_{2}^{2}-2r_{1}r_{2}\cos\varphi \left(t\right)}{{\left(1-r_{1}r_{2}\right)}^{2}+4r_{1}r_{2}\sin^{2}(\varphi (t)/2)},$$

where *r*_1_ denotes the reflectivity of the outgoing end-face of the fiber, *r*_2_ denotes the reflectivity of the object to be measured, and $\varphi \left(t\right)$ represents the phase to be measured.

As shown on the right side of Figure 2, the F-P cavity is a low-fineness cavity when *r*_1_ and *r*_2_ are small, and the multibeam interference model of the fiber optic F-P micro-sensing probe can be approximated using a two-beam interference model; therefore, Equation (4) can be simplified in the following format:

(5) $$I_{\mathrm{FP}}=I_{0}\cdot (r_{1}^{2}+r_{2}^{2}-2r_{1}r_{2}\cos\varphi \left(t)\right),$$

However, the multibeam interference that exists in the F-P cavity is ignored due to the equivalent treatment in the measurement principle, which results in non-negligible errors. In Figure 2, the blue and red arrows indicate the reference and measurement lights, respectively. The reference light’s vibration equation for the fiber F-P microprobe interferometer can be expressed as:

(6) $$E_{r}=A_{r}\exp\left[i\left(\omega t+\varphi_{r}\right)\right],$$

where $A_{r}$ denotes the reference light’s amplitude, ω represents the reference light’s angular frequency, and $\varphi_{r}$ denotes the reference light’s initial phase.

Due to the motion of the measurement object, the vibration equation of the light exiting the fiber after one reflection from the target inside the cavity is expressed as:

(7) $$E_{m}=A_{m}\exp\left[i\left(\omega t+\varphi_{0}+\varphi_{m}\right)\right],$$

where $A_{m}$ denotes the amplitude of the light measured by one reflection, ω represents the angular frequency of the light measured by the fiber, $\varphi_{0}$ denotes the initial phase, and $\varphi_{m}$ represents the phase corresponding to the displacement to be measured.

A number of reflection phenomena can be seen in Figure 2. After the 1st reflection of the light, part of it goes through the optical fiber, whereas another part transmits through the end-face of the optical fiber, re-incidences to the target, and forms the basis of the subsequent reflections.

The vibrational equation of the light formed in the cavity after *n* reflections is:

(8) $$E_{m\_\mathrm{all}}=\sum_{j=1}^{n}A_{m\_j}\exp\left[i\left(\omega t+\varphi_{0}+j\varphi_{m}\right)\right],$$

The interference signal obtained from the reference light and the reflected measurement light interfere with each other in the fiber optic F-P microprobe interferometer, mathematically represented by the following expression:

(9) $$I\propto Re\left[\left(E_{m\_\mathrm{all}}+E_{r}\right)\cdot {\left(E_{m\_\mathrm{all}}+E_{r}\right)}^{*}\right],$$

According to Equation (9), the interference signal produced using an F-P type interference probe can be represented by the following expression:

(10) $$\begin{array}{ll} I & =A+B_{1}\cos(C\cos\mathrm{wt}+\varphi_{m}{)+B}_{2}\cos(2C\cos\mathrm{wt}+2\varphi_{m})+\ldots \\ & +B_{n-1}\cos((n-1)\cdot \cos\mathrm{wt}+(n-1)\varphi_{m}{)+B}_{n}\cos(n\cdot \cos\mathrm{wt}+n\varphi_{m}) \end{array},$$

where *A* denotes the DC component’s amplitude of the signal, *B*_n_ represents the *n*th AC signal amplitude, and *w* denotes the angular frequency of the sinusoidally modulated signal.

According to Equation (10), the F-P interference generates additional multiple harmonic signals compared to the two-beam interference, and the quadrature components of the interference signal are obtained through PGC demodulation as follows:

(11) $$S_{1}=B_{1}J_{1}(C)\sin\varphi_{m}{+B}_{2}J_{1}(2C)\sin2\varphi_{m}+\ldots {+B}_{n}J_{1}(n\cdot C)\sin n\varphi_{m},$$

(12) $$S_{2}=B_{1}J_{2}(C)\cos\varphi_{m}{+B}_{2}J_{2}(2C)\cos2\varphi_{m}+\ldots {+B}_{n}J_{2}(n\cdot C)\cos n\varphi_{m},$$

**Figure 3:**
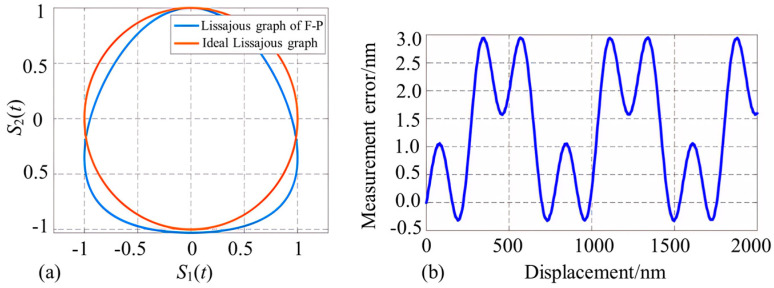
Demodulation results associated with the interferometric signals of the fiber optic F-P microprobe (note: (**a**) represents the Lissajous graph of the orthogonal signals, and (**b**) represents the displacement demodulation error).

The corresponding Lissajous graphs are plotted with *S*_1_ and *S*_2_ as the horizontal and vertical coordinates, respectively, and it can be seen that the Lissajous graphs obtained from the fiber optic F-P interferometric probe are not in the conventional ellipse form (Figure 3a).

Next, the demodulation error obtained from the F-P sensing probe is simulated, and the effective reflectivity of the object’s reflective surface in the F-P cavity of the microprobe is set to be 0.6 considering the light intensity attenuation during the specular reflection process (Figure 3b). According to this figure, the maximum error value reaches 3 nm; therefore, it is difficult to achieve the nanoscale accuracy measurement requirements using this interferometric probe.

Therefore, we propose to use a microprobe structure based on the fiber optic Michelson interference principle divided into collimated and convergent types. Because it is a double-beam interference in principle, the interference signal obtained using this probe type does not have nanometer-scale error, making it possible to measure sub-nanometer precision measurements using this fiber microprobe laser interferometer.

### 2.3. Design of a Collimated Michelson Micro-Sensing Probe

**Figure 4:**
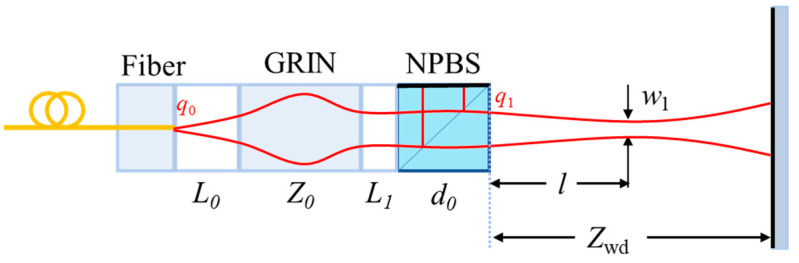
Composition diagram of collimated Michelson micro-sensing probe (note: *L*_0_ denotes the length of the air gap after the fiber end-face, *Z_0_* denotes the length of the GRIN lens, *L*_1_ denotes the length of the air gap between the GRIN lens and the NPBS, *d*_0_ denotes the length of the NPBS, *l* denotes the beam waist position, *w*_1_ denotes the waist size, *Z_wd_* denotes the working distance, *q*_0_ denotes the Gaussian beam on the fiber outgoing end-face, and *q*_1_ denotes the Gaussian beam after passing through the microprobe. NPBS: nonpolarized beam splitter; GRIN: Gradient Index lens).

The proposed collimated Michelson microprobe (shown in Figure 4) consists of a single-mode fiber pigtail, an air gap, a gradient refractive index (GRIN) lens, and a nonpolarized beam splitter (NPBS).

The output light field of a single-mode fiber can be approximated as a Gaussian distribution of the waist at the fiber end-face, and its waist size *w*_0_ is determined using the fiber mode field diameter (MFD). After the Gaussian beam is collimated by the GRIN lens and split by the beam splitter in equal proportions, one beam of light returns to the GRIN lens as the reference light through the NPBS total reflection surface, and the other beam of light propagates in the air for *Z*_wd_ and is then reflected back to the GRIN lens by the reflector to couple with the reference light in order to produce an interference signal.

Next, the Gaussian beam complex size of curvature *q*-parameter transformation and the *ABCD* transmission matrix are used to construct a model of the relationship between the parameters of each element of the probe and the size of the output beam waist and the working distance. The *q*-parameters are expressed as follows:

(13) $$\frac{1}{q\left(z\right)}=\frac{1}{R\left(z\right)}-i\frac{\lambda }{\pi w^{2}\left(z\right)},$$

where *R*(*z*) is the curvature size of the isophase plane of the Gaussian beam located at *z*, *w*(*z*) is the spot size of the isophase plane of the Gaussian beam located at *z*, and *λ* denotes the light wavelength.

The Gaussian beam at the fiber end-face is $q_{0}=i\frac{\pi n_{0}w_{0}^{2}}{\lambda }=if_{0}$, and *n*_0_ is the air gap refractive index. The *ABCD* transmission matrix M_0_ of the first air gap is $\left[\begin{array}{cc} 1 & L_{0}\\ 0 & 1 \end{array}\right]$. The collimating lens uses a radial gradient refractive index lens, the refractive index changes along the direction perpendicular to the optical axis, and the refractive index distribution equation is $n\left(r\right)=n_{1}\left(1-\frac{Ar^{2}}{2}\right)$, where *n*_1_ is the refractive index on the optical axis, $b=\sqrt{A}$ is the self-focusing constant, and the transmission matrix M_1_ of the GRIN lens is $\left[\begin{array}{cc} \cos\sqrt{A}Z_{0} & \frac{\sin\sqrt{A}Z_{0}}{n_{1}\sqrt{A}}\\ -n_{1}\sqrt{A}\sin\sqrt{A}Z_{0} & \cos\sqrt{A}Z_{0} \end{array}\right]$. The transmission matrix M_2_ of the second air gap is $\left[\begin{array}{cc} 1 & L_{1}\\ 0 & 1 \end{array}\right]$, the transmission matrix M_3_ of the NPBS is $\left[\begin{array}{cc} 1 & \frac{d_{0}}{n_{2}}\\ 0 & 1 \end{array}\right]$, and *n*_2_ is the refractive index of the NPBS.

The *q*-parameter of the light after passing through the microprobe is obtained from the *ABCD* transformation law for Gaussian beams:

(14) $$q_{1}=\frac{Aq_{0}+B}{Cq_{0}+D},$$

where *A*, *B*, *C*, and *D* represent the four parameters of the transmission matrix M, respectively, and M = M_3_ × M_2_ × M_1_ × M_0_.

Based on the above analysis, the expressions for the output beam waist size and probe working distance are obtained as:

(15) $$w_{1}=\sqrt{\frac{f_{0}\lambda }{D^{2}+\pi C^{2}f_{0}^{2}}},$$

(16) $$Z_{wd}=-2\frac{\left(BD+ACf_{0}^{2}\right)}{D^{2}+C^{2}f_{0}^{2}},$$

The output spot size versus probe working distance is given by:

(17) $$w(Z_{wd})=w_{1}\sqrt{1+{\left(\frac{\lambda Z_{wd}}{\pi w_{1}^{2}}\right)}^{2}},$$

**Figure 5:**
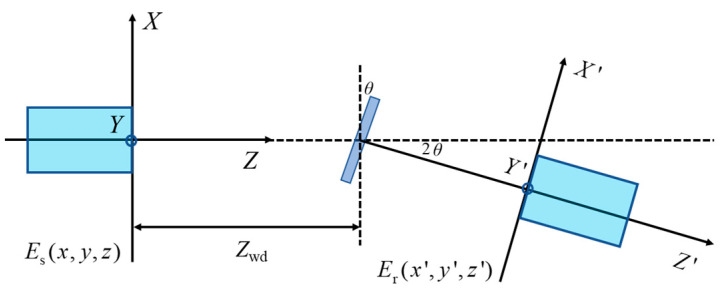
Output optical coupling model of symmetrically equivalent microprobe (note: *E*_s_ denotes the output optical field in a coordinate system established at the outgoing end-face of the probe, and *E*_r_ represents the effective received optical field in coordinates established at the equivalent probe end-face).

The sensitivity of the fiber optic sensing probe to changes in object inclination and working distance is investigated by translating and tilting the reflector. When the reflective target has *θ* inclination, the light will become an off-axis Gaussian beam after passing through the reflective target, which has a 2*θ* angle with the optical axis of the system. This makes it difficult to simulate the transmission path of the Gaussian beam. As shown in Figure 5, the symmetric equivalent fiber receiving probe is established with the reflector as the axis following a method borrowed from the literature [18]. The beam interference can be equated to the signal light coupling from the original probe and the intrinsic reference light from the equivalent probe at the end-face of the equivalent probe.

A coordinate system is established with the fiber optic probe outgoing end-face as well as the equivalent probe end-face in order to obtain the expressions for the reference and measured lights’ electric fields. The optical coupling efficiency of the fiber optic sensing probe can be given by the overlapping integrals of the output photoelectric field and the intrinsic mode field of the equivalent probe at the equivalent probe end-face.

In the absence of declination of the object to be measured, the power coupling efficiency of the fiber optic interferometric probe with respect to the working distance is obtained as follows, based on a relationship between the coordinate systems established by the two probe end-faces (*x* = *x*′, *y* = *y*′, *z* = *z*′ + 2*Z*_wd_):

(18) $$\eta_{1}=\frac{4f^{4}[f^{2}{+(2Z}_{\mathrm{wd}}{-l)}^{2}](f^{2}{+l}^{2})}{f^{4}{\left[{2f}^{2}{+(2Z}_{\mathrm{wd}}{-l)}^{2}{+l}^{2}\right]}^{2}{+w}_{1}^{4}k^{2}{\left[f^{2}{-(2Z}_{\mathrm{wd}}-l)l\right]}^{2}{{(Z}_{\mathrm{wd}}-l)}^{2}},$$

where *k* is the wave number and *f* is the confocal parameter of the output Gaussian beam, described as $f=\frac{\pi nw_{1}^{2}}{\lambda }$.

Setting the reflector at the position of the outgoing beam waist (i.e., *Z*_wd_ = *l*), there exists an inclination angle *θ* of the reflector, according to the relationship of the coordinate system established between the actual and equivalent probes’ end-faces (i.e., *x* = *x*′cos2*θ* − *z*′sin2*θ* − *Z*_wd_ sin2*θ*, *y* = *y*′ and *z* = *x*′sin2*θ* + *z*′cos2*θ* + *Z*_wd_ + *Z*_wd_cos2*θ*). The power coupling efficiency of the fiber optic probe is obtained as:

(19) $$\eta_{2}=\exp[-\sin^{2}\theta (\frac{l^{2}f^{2}}{w_{1}^{2}{(l}^{2}{+f}^{2})}+\frac{w_{1}^{2}k^{2}f^{2}}{{4(l}^{2}{+f}^{2})})],$$

The interfering signal quality is also measured on the basis of the interferometric fringe contrast, which is proportional to the signal-to-noise ratio of the measured signal and is also related to the displacement-sensing sensitivity. According to Equation (2), the fringe contrast is given by the following equation:

(20) $$V=\frac{I_{M}-I_{m}}{I_{M}+I_{m}}=\frac{2\sqrt{I_{r}I_{s}}}{I_{r}+I_{s}}=\frac{B}{A},$$

where *I*_M_ is the interferometric light intensity maximum, *I*_m_ is the interferometric light intensity minimum, *I*_r_ = |*E*_r_|^2^, and *I*_s_ = |*E*_s_|^2^.

From the above analysis, it was found that several parameters affect the final output beam’s waist position, waist size, and spot size, such as the distance between the single-mode fiber pigtail and the GRIN lens *(L*_0_), the self-focusing constant *b*, the length *Z*_0_, the central refractive index of the GRIN lens *n*_1_, the distance between the GRIN lens and the NPBS *L*_1_, the dimensions of the NPBS, *d*_0_, and the material *n*_2_. The beam waist position and size directly determine the beam coupling efficiency, *η*, and interferometric pattern contrast, *V*, which also indirectly determine the probe working distance, *Z*_wd_, and tolerance angle *θ*.

Considering the equations presented so far, the structural parameters of each element are designed in the following section. It is assumed that for the GRIN lens, *b* is 0.595 mm^−1^, *n*_1_ is 1.591, and *Z*_0_ is 5.68 mm. The control variable method is also used to investigate the effects of each factor on the probe working distance *Z*_wd_ and tolerance angle *θ* in the collimated microprobe structure. The target for these objective functions is to meet the coupling efficiency of more than 20% while having a contrast ratio of more than 0.8 that is continuous.

**Figure 6:**
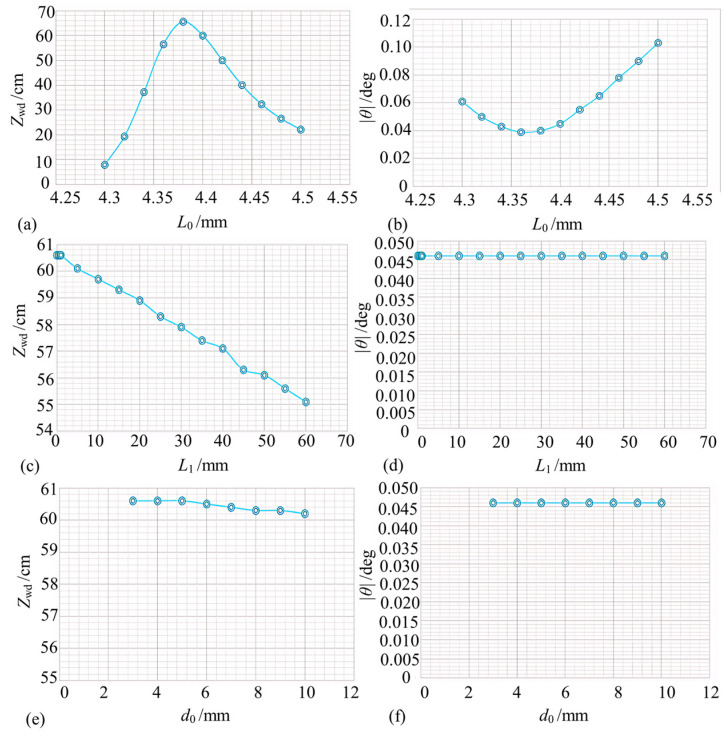
Parameter design for collimated Michelson microprobe (note: (**a**,**b**) indicate the effect of air gap length *L*_0_ on *Z*_wd_ as well as *θ*; (**c**,**d**) indicate the effect of the distance between GRIN and NPBS *L*_1_ on *Z*_wd_ and *θ*; and (**e**,**f**) indicate the effect of NPBS length on *Z*_wd_ and *θ*).

Firstly, the effect of the air gap length *L*_0_ on the working distance *Z*_wd_ and tolerance angle *θ* is explored. Taking *L*_1_ = 0 mm, *d*_0_ = 3 mm, and *n*_2_ = 1.50091 (N-bk7), and assuming the output of single-mode fiber to be *w*_0_ = 5.2 μm and *λ* = 1.550 μm, the relationship between *L*_0_ and the working distance *Z*_wd_ as well as tolerance angle *θ* is obtained by constantly varying *L*_0_ (Figure 6a,b). The collimated microprobe can work normally when *L*_0_ is in the range of 4.3–4.5 mm (Figure 6a). With an increasing *L*_0_, the probe working distance *Z*_wd_ first increases, reaches a maximum value of 65.6 cm at *L*_0_ = 4.38 mm, and then decreases. Moreover, with an increasing *L*_0_, the probe tolerance angle |*θ*| first becomes smaller, reaches a minimum value of 0.039° at *L*_0_ = 4.36 mm, and then becomes larger (Figure 6b). In summary, the distance from the single-mode fiber pigtail to the GRIN lens *L*_0_ significantly affects the probe’s overall performance. It is difficult to ensure that a sufficiently large working distance is obtained while obtaining a sufficient probe tolerance angle. Considering the compromise, *L*_0_ = 4.4 mm is finally taken.

Next, the effect of the distance between GRIN and NPBS *L*_1_ on the working distance *Z*_wd_ and tolerance angle *θ* is explored. Taking *L*_0_ = 4.4 mm, *d*_0_ = 3 mm, and *n*_2_ = 1.50091 (N-bk7), the value of *L*_1_ is continuously changed to obtain the variation curves for the working distance and tolerance angle (Figure 6c,d). With an increasing *L*_1_, the probe working distance *Z*_wd_ slightly decreases; therefore, *L*_1_ slightly affects the probe working distance within the variation range of 0–60 mm. From Figure 6d, *L*_1_ also minimally affects the probe tolerance angle within the variation range of 0–60 mm. Therefore, *L*_1_ = 0 mm is selected considering the machining process taking into account the overall dimensions of the probe.

Finally, the effect of NPBS dimension *d*_0_ on the working distance *Z*_wd_ and tolerance angle *θ* is explored considering *L*_0_ = 4.4 mm, *L*_1_ = 0, and *n*_2_ = 1.50091 (N-bk7). As seen in Figure 6e,f, NPBS size *d*_0_ has no effect on the probe working distance *Z*_wd_ or tolerance angle *θ*. To match the GRIN lens size as well as the NPBS production cost, *d*_0_ = 3 mm is chosen.

### 2.4. Design of a Convergent Michelson Micro-Sensing Probe

**Figure 7:**
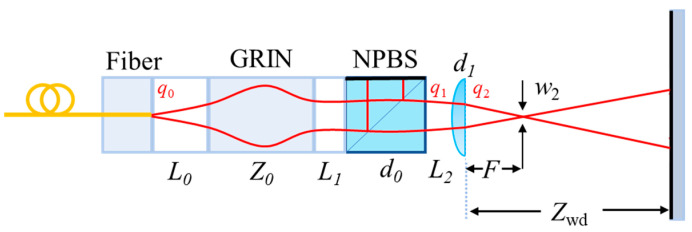
The schematic composition diagram of the converging Michelson microprobe (note: *L*_2_ is the distance from the NPBS to the converging lens, *F* is the focal length of the converging microprobe, *w*_2_ is the focusing waist size, *Z_wd_* is the working distance of the converging microprobe, and *d*_1_ is the thickness of the lens center).

As shown in Figure 7, the converging microprobe design adds a converging lens to the collimated Michelson microprobe structure, which converges the measurement light onto the target to be measured and allows the microprobe tolerance angle to be increased.

The air gap transmission matrix between the NPBS and converging lens M_4_ is $\left[\begin{array}{cc} 1 & L_{2}\\ 0 & 1 \end{array}\right]$, and the Gaussian beam transmission matrix of the converging lens M_5_ is $\left[\begin{array}{cc} 1 & 0\\ 0 & n_{3} \end{array}\right]\left[\begin{array}{cc} 1 & d_{1}\\ 0 & 2 \end{array}\right]\left[\begin{array}{cc} 1 & 0\\ \frac{n_{3}-1}{-n_{3}R_{1}} & \frac{1}{n_{3}} \end{array}\right]$, where *n*_3_ is the refractive index of the lens, *d*_1_ is the lens center thickness, and *R*_1_ is the curvature size of the lens.

The final output beam waist parameter is affected not only by the collimated structure variables but also by several new variables associated with the converging probe, such as the distance between the NPBS and converging lens *L*_2_, the center thickness of the single lens *d*_1_, and the size of the curvature *R*_1_ and the refractive index *n*_3_. All of these variables affect the probe working distance *Z*_wd_ and tolerance angle *θ*.

**Figure 8:**
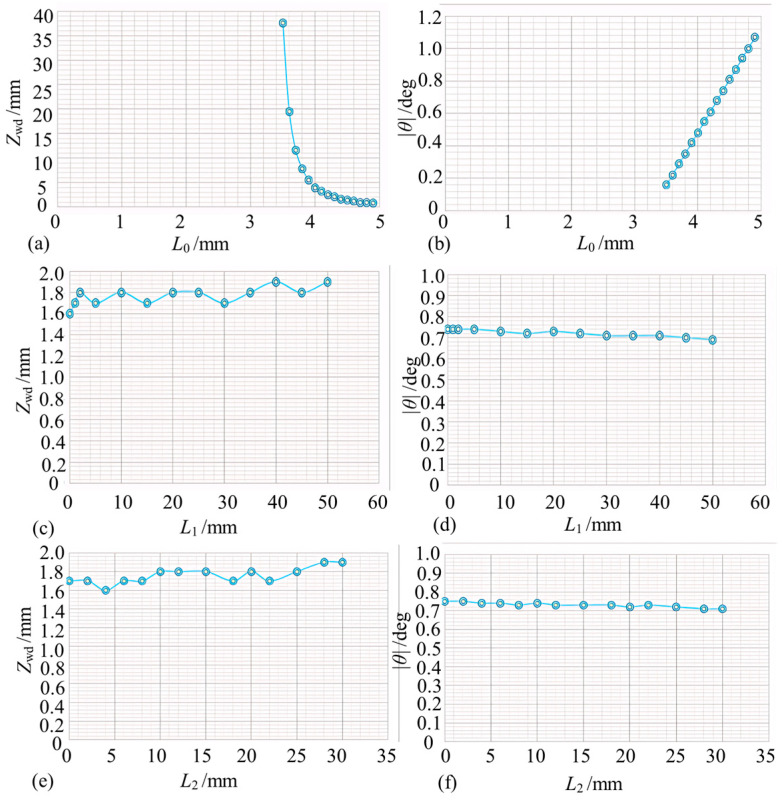
Convergent Michelson microprobe parameter design (note: (**a**,**b**) denote the effect of air gap length *L*_0_ on *Z*_wd_ and *θ*; (**c**,**d**) denote the effect of the distance between GRIN and NPBS *L*_1_ on *Z*_wd_ and *θ*; and (**e**,**f**) denote the effect of the distance between NPBS and converging lens *L*_2_ on *Z*_wd_ and *θ*).

For the converging microprobe structure, the effects of each important factor on the probe working distance *Z*_wd_ and tolerance angle *θ* are investigated using the control variable method assuming a GRIN lens with *b* of 0.595 mm^−1^, *n*_1_ of 1.591, and *Z*_0_ of 5.68 mm. Firstly, the effect of air gap length *L*_0_ on working distance *Z*_wd_ and tolerance angle *θ* is explored assuming *L*_1_ = 0, *d*_0_ = 3 mm, *n*_2_ = 1.50091 (N-bk7), and *L*_2_ = 4 mm (Figure 8a,b). The converging microprobe works properly when *L*_0_ is in the range of about 3.5–5 mm. Increasing *L*_0_ rapidly declines the probe operating range *Z*_wd_ while linearly increasing the probe tolerance angle |*θ*|. It is concluded that keeping *L*_0_ in the range of 4–4.5 mm puts the working range *Z*_wd_ and tolerance angle *θ* in a large enough variation range.

The effect of the distance between GRIN and NPBS *L*_1_ on the working distance *Z*_wd_ and tolerance angle *θ* is then investigated assuming *L*_0_ = 4.4 mm and *L*_2_ = 4 mm (Figure 8c,d). In addition, the effect of *L*_2_ on *Z*_wd_ and *θ* is obtained assuming *L*_0_ = 4.4 mm and *L*_1_ = 0 (Figure 8e,f). From these figures, it is concluded that *L*_1_ and *L*_2_ have an insignificant impact on the probe’s performance parameters.

## 3. Results

### 3.1. Experimental Setup

According to Figure 1, the two Michelson structure microprobe sensors designed above were used to build the overall fiber laser interferometer system. The laser source used in the experiments was a distributed feedback laser (DFB) (DFB PRO BFY, Toptica, Gräfelfing, Germany), which was modulated by a high-speed sinusoidal signal. The object under test was a P-733.3DD three-dimensional piezoelectric ceramic nano-displacement stage (Physik Instrument, Karlsruhe, Germany) with an ultimate displacement resolution of 0.4 nm, which can be programmed to achieve different forms of motion. The optoelectronic conversion of the interfering signals was implemented using an APD (APD430C, Thorlab, Newton, NJ, USA) converted to a digital signal using a 16-bit digital-to-analog converter (ADC). The phase demodulation occurred using the PGC algorithm, and the final demodulated displacements were sent to a personal computer for display via Universal Serial Bus (USB). All of the modulation and demodulation modules were operated in a Field Programmable Gate Array (FPGA). All experiments were performed in an ultra-precision clean laboratory (ΔT = 0.01 °C/10 min) on an air-float table.

### 3.2. Experimental Performance Tests for Collimated Michelson Microprobes

#### 3.2.1. Simulation Results Associated with Collimated Microprobes

Numerical simulation of the constructed collimated microprobe was carried out using the model presented in Section 2.3 assuming a Gaussian beam waist size $w_{0}=5.2\times 10^{-6}$ m, an air gap length $L_{0}=4.4\times 10^{-3}$ m, a GRIN lens length $Z_{0}=5.679\times 10^{-3}$ m, a self-focusing constant $\sqrt{A}=0.595$ mm^−1^, a refractive index $n_{1}=1.591$, an NPBS length $d_{0}=3\times 10^{-3}$ m, and a refractive index $n_{2}=1.50091$. The microprobe beam waist position was obtained at *l* = 16.7 cm with size *w*_1_ = 725 μm.

**Figure 9:**
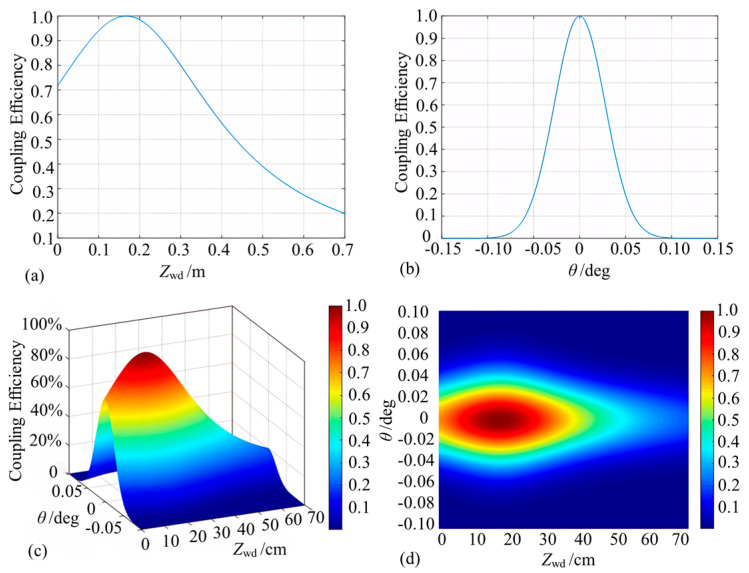
Simulation results of coupling efficiency of collimated micro-sensing probes (note: (**a**,**b**) denote the coupling efficiency curves for working distance *Z*_wd_ and mirror inclination alone, respectively, and (**c**,**d**) denote the coupling efficiency surfaces for the combined effect of working distance and angle).

Firstly, the power coupling efficiency of the fiber optic sensing probe was calculated based on the working distance *Z*_wd_ and the deflection angle of the reflector. According to Figure 9a, the probe receives a theoretically identical light wave field with an intrinsic light field when the working distance is at the position of the output beam waist, which caused 100% coupling efficiency. The power coupling efficiency versus deflection angle was calculated when the reflector was at the output beam waist position (Figure 9b). When the beam was vertically incident to the reflector, the optical power returned to the probe was the largest. The optical power captured by the microprobe decreased rapidly with the increase in the deflection angle when there was a deflection angle between the normal value of the reflecting surface and the incident beam. The power coupling efficiency was about 19% when the deflection angle of the reflector was ±0.05°.

In order to investigate the combined effects of working distance and mirror inclination on the power coupling efficiency of the probe, the three-dimensional plot presented in Figure 9c can be used. Only when the working distance was located at the beam waist and positive incidence could the fiber probe fully receive the reflected light field, and the current maximum power coupling efficiency was not 100% if any of these parameters were not in the optimal condition. When *Z*_wd_ was in the range of 8 cm to 26 cm and *θ* was changing between −0.01° and 0.01°, the power coupling efficiency stayed above 80%, and the fiber probe was more sensitive to the change in the reflector tilt angle (Figure 9d).

**Figure 10:**
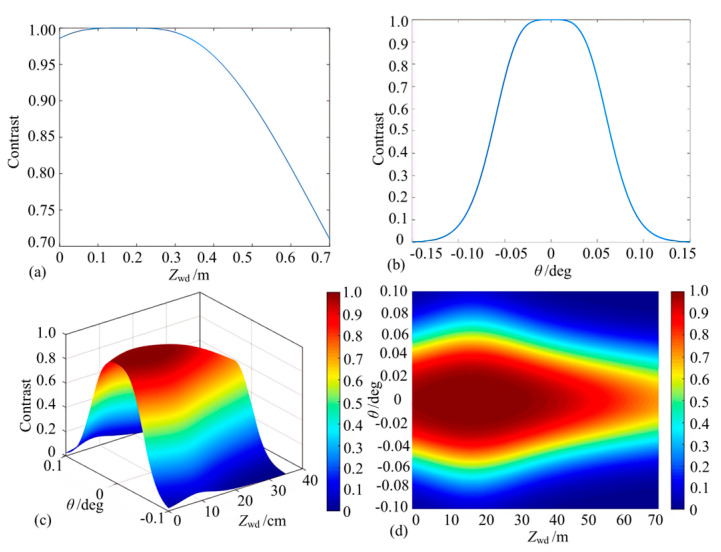
Simulation of the contrast of collimated micro-sensing probes (note: (**a**,**b**) represent the contrast variation under the effect of working distance and angle alone, and (**c**,**d**) represent the contrast surface plots under the combined effect of the two factors).

The interferometric signal contrast of the equivalent receiving fiber probe end-face was next simulated as a function of the working distance and reflector inclination (Figure 10a and Figure 10b, respectively). According to these figures, the interferometric fringe contrast varies more gently with respect to the working distance and reflector inclination angle and maintains a better contrast within a range when compared with the simulation results associated with the microprobe coupling efficiency. Then, both the working distance and mirror inclination angle are varied simultaneously (Figure 10c,d). The contrast is characterized by a gentle change, and the interference fringe contrast is theoretically greater than 0.8 in the range of *Z*_wd_ = 0 cm to 50 cm and *θ* = −0.04° to 0.04°.

#### 3.2.2. Experimental Testing of Collimating Probes

**Figure 11:**
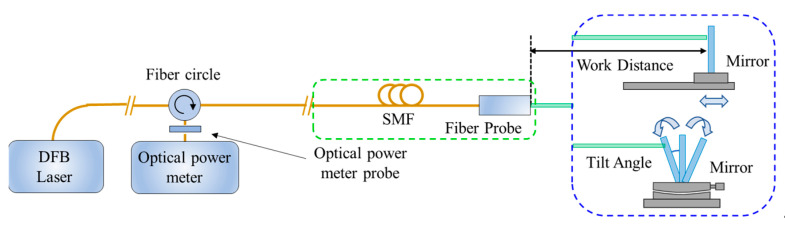
Schematic diagram of probe performance testing system.

**Figure 12:**
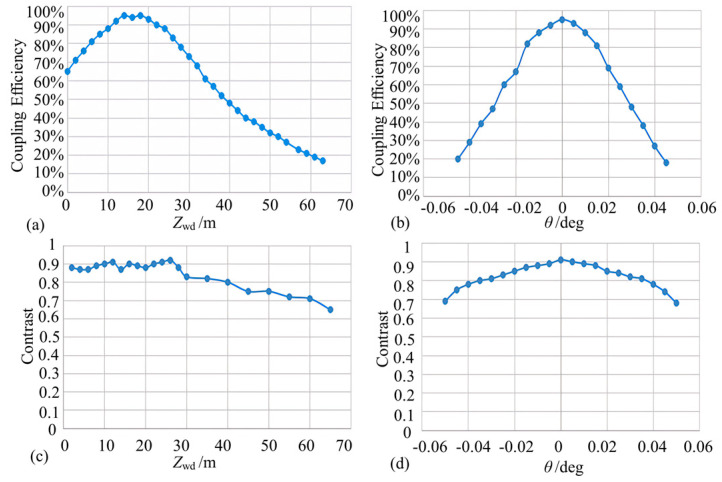
Test results of collimated micro-sensing probes (note: (**a**,**b**) denote the effects of different working distances and mirror deflection angles on the coupling efficiency, respectively, and (**c**,**d**) denote the corresponding contrast variation curves).

The performance index of the assembled probe was tested (Figure 11). For the calculation of coupling efficiency, an optical power meter was used to measure the direct light power out of the probe as a reference, and the optical power magnitude reflected back was observed and recorded at different mirrors’ working distances and tolerance angles. The return light coupling efficiency as a function of working distances and tilt angle was obtained (Figure 12a,b). In the range of a 0–70 cm distance, the coupling efficiency first increases, reaches a maximum value of about 94% in the range of 14–20 cm, and then decreases. In the ±0.05° angle working range, the coupling efficiency curve has parabolic symmetry, and the tilt angle has a greater impact on the collimated microprobe coupling efficiency.

To calculate the interference fringe contrast, the interference light coupled back under different working distances and mirror angles was connected to an oscilloscope after passing through the photoelectric converter in order to observe the DC magnitude as well as AC components (Figure 12c and Figure 12d, respectively). When the working distance is at 0–40 cm, the interfering signal contrast can reach more than 0.8, which belongs to the more ideal range. The tilt angle contrast trend is similar to that of the coupling efficiency, and the attenuation is extremely fast upon increasing the tilt angle. Therefore, the working distance and probe tolerance angle can be up to 40 cm and ±0.03°, respectively, for collimated Michelson microprobe structures.

### 3.3. Experimental Performance Tests for Convergent Michelson Microprobes

#### 3.3.1. Simulation Results Associated with Convergent Microprobes

The relationship between the microprobe working distance and the outgoing spot was constructed as a model based on the *ABCD* transmission transformation law associated with the complex curvature parameter *q* size. Assuming a distance parameter of *L*_2_ = 2 mm and a converging lens parameters of *F* = 20 mm, *n*_3_ = 1.5176, *d*_1_ = 2.5 mm, and *R*_1_ = 10 mm, the beam waist position and size were obtained at 17.60 mm and *w*_2_ = 22.363 μm, respectively.

**Figure 13:**
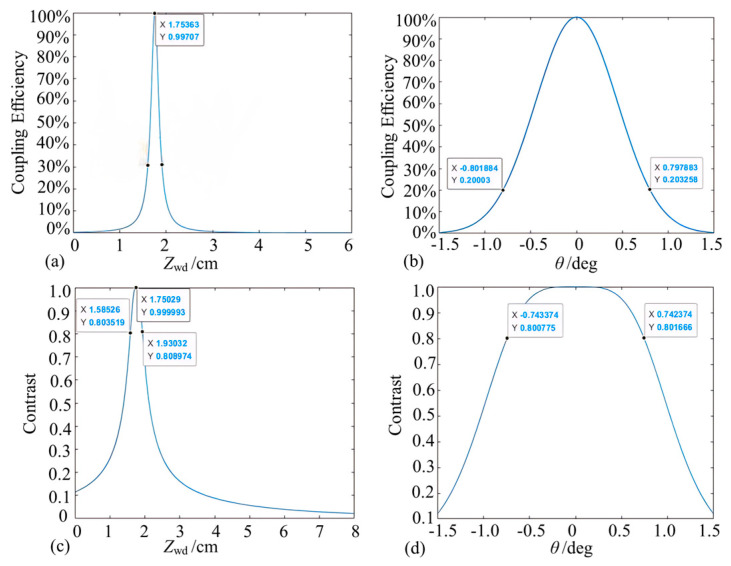
Simulation of convergent micro-sensing probes (note: (**a**,**b**) denote the coupling efficiency variations, and (**c**,**d**) denote the contrast variations).

Similarly to Section 3.2.1, theoretical models were developed for convergent microprobe coupling efficiency and interference fringe contrast as functions of probe working distance and reflector inclination (Figure 13). According to Figure 13a, the coupling efficiency is close to 100% when the working distance is near the output waist position. When the working distance deviates from the waist position, the coupling efficiency decreases sharply. As shown in Figure 13b, the reflector deflection angle should be changed to obtain power coupling efficiency and the deflection angle when the reflector is located near the output beam waist position. When the beam is vertically incident to the reflector, the optical power returned to the probe is the greatest. When the reflecting surface is normal and the incident beam exists at a deflection angle, the optical power captured by the microprobe decreases rapidly with an increase in the deflection angle, and the power coupling efficiency is about 20% when the deflection angle of the reflector is ±0.8°.

As shown in Figure 13c,d, the contrast change trend is similar to that of the coupling efficiency. When the working distance is at 1.75 ± 0.18 cm and the deflection angle of the reflector is within ±0.74°, the contrast can reach more than 0.8, meeting the working requirements.

#### 3.3.2. Experimental Testing of Convergent Probes

**Figure 14:**
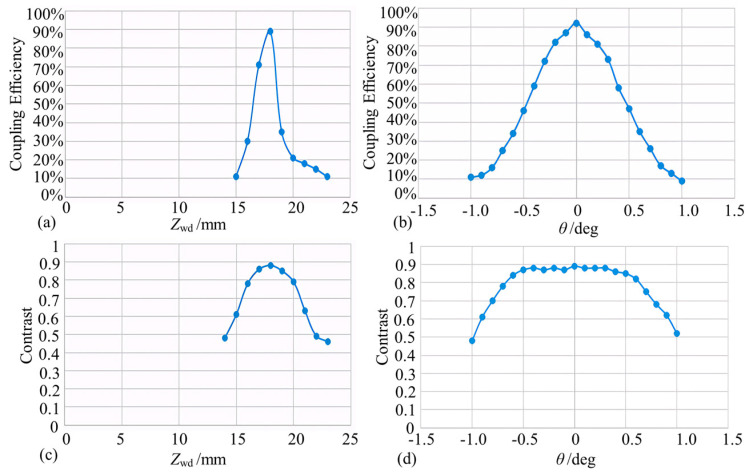
Converging lens working distance and tolerance angle test (note: (**a**,**b**) indicate the effect of different working distances and mirror deflection angles on the coupling efficiency, respectively, and (**c**,**d**) indicate the corresponding contrast variation curves).

With the same test method as that discussed in Section 3.2.2, the return light coupling efficiency and convergence probe contrast as a function of working distance and tilt angle are calculated (Figure 14). In the working distance range of 17.6 mm ± 0.5 mm, the coupling efficiency increases first, reaches the maximum coupling efficiency, and then decreases. The maximum coupling efficiency of about 90% appears near the waist. As for the tilt angle, the coupling efficiency of 50% or more is regarded as the usable range, and the tolerance angle range is about ±0.5°.

As shown in Figure 14c,d, when the working distance is at 17.6 mm ± 1.5 mm, the interference signal contrast can reach more than 0.8. The tilt angle contrast trend is similar to that of the tilt angle coupling efficiency, and the attenuation is extremely fast with the increase of the tilt angle. It is then concluded that for convergent Michelson microprobe structure, the working distance and reflector tolerance angle could reach 17.6 mm ± 0.5 mm and ±0.5°, respectively.

### 3.4. Displacement Resolution Test Results

**Figure 15:**
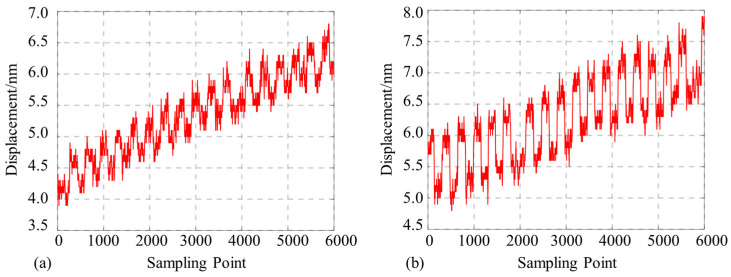
Displacement measurement resolution test for the proposed probe (note: (**a**) indicates the results of displacement demodulation at 0.4 nm and (**b**) at 0.8 nm).

The displacement stage was controlled to move back and forth for 0.8 nm and 0.4 nm, i.e., to perform the test with 0.8 nm and 0.4 nm resolutions. The probe was replaced and tested again, and the results are shown in Figure 15.

The 0.8 nm and 0.4 nm displacement demodulation results are clearly recognizable using our proposed probe. Although there is a directional drift in these measurements due to the displacement stage accuracy as well as the ambient temperature, this can be reduced by further improving the environmental stability at a later stage. Therefore, our proposed Michelson micro-sensing structure enables the fiber microprobe laser interferometer to achieve a sub-nanometer-scale displacement measurement accuracy of 0.4 nm.

## 4. Discussion and Conclusions

Today’s research on fiber optic micro-sensing probes mainly focuses on the interferometric structure of F-P cavities, a model that approximates multibeam interference as two-beam interference for sensing. It does not take into account the measurement principle error caused by multibeam reflection. In order to achieve sub-nanometer-level precision displacement measurement for a micro-sensing probe, this paper first described the general measurement principle of ultra-precision laser interference of fiber optic microprobe and then derived and simulated the limitations associated with a fiber optic Fabry–Perot interferometer. Subsequently, a sensing method and model for a Michelson microprobe structure was investigated based on a self-focusing lens. The mapping relationship between the sensing probe design parameters and measurement distance and tolerance angle was established. Parametric simulation was carried out according to the proposed model, and the proper design for the collimated Michelson fiber microprobe was then realized. In addition, a convergent Michelson microprobe design was completed to meet the requirements of large-tolerance angular measurements.

The final probe design achieved the following properties: the collimated probe can reach a working distance of 40 cm and a tolerance angle of ±0.03° under the premise of a coupling efficiency of 50% or more and a contrast ratio of 0.8. The convergent probe can reach a tolerance angle of ±0.5° at a beam waist of 17.6 ± 0.5 mm. The above two probes are suitable for long-distance and large tolerance angle displacement measurement scenarios. By applying the above probes to the fiber laser interferometer, the final displacement resolution can reach 0.4 nm. In the future, we will focus on researching micro-sensing probes with longer working distances and larger tolerance angles to realize large-range and high-precision displacement measurement.

## Data Availability

The data that support the findings of this study are available from the authors upon reasonable request.
